#  A Continuous Transversus Abdominis Plane Block Decreases Hospital Length of Stay Compared to Thoracic Epidural Analgesia After Open Radical Cystectomy Surgery: A Retrospective Study

**DOI:** 10.5812/aapm-143354

**Published:** 2024-01-17

**Authors:** Sanaz Beig Zali, Rachel Steinhorn, Vivian Hu, Linda Hung, Francis McGovern, Farbod Alinezhad, Tammer Yamany, Thomas Anthony Anderson, A. Sassan Sabouri

**Affiliations:** 1 Department of Anaesthesia, Critical Care and Pain Medicine, Massachusetts General Hospital, Boston, MA, USA; 2 Department of Anaesthesia, Critical Care and Pain Medicine, Massachusetts General Hospital, Harvard Medical School, Boston, MA, USA; 3 San Diego Medical Center, San Diego, CA, USA; 4 University of Calgary, Calgary, Canada; 5 Department of Urology, Massachusetts General Hospital, Boston, MA, USA; 6 Northeastern University, Boston, MA, USA; 7 Lenox Hill Hospital, New York, NY, USA; 8 Department of Anesthesiology, Perioperative and Pain Medicine, Stanford University, Stanford, CA, USA; 9 Department of Anesthesia, Shahid Beheshti University of Medical Sciences, Tehran, Iran

**Keywords:** Epidural Analgesia, Thoracic, Transversus Abdominis Plane Block, Cystectomy, Urinary Bladder, Analgesia, Patient-Controlled, Length of Stay, Ambulation

## Abstract

**Background:**

Poorly managed postoperative pain can prolong hospital stays and increase the risk of complications in patients undergoing open radical cystectomy (ORC). Despite strong support from the clinical guidelines for using surgical site-specific peripheral regional anesthetic techniques and neuraxial analgesia, their effects on postoperative outcomes are unclear.

**Objectives:**

This study aims to fill the above knowledge gap by comparing thoracic epidural analgesia (TEA) and continuous transversus abdominis plane (CTAP) blocks in ORC patients.

**Methods:**

In this retrospective observational study, we conducted chart reviews at a quaternary care academic hospital in Boston, Massachusetts, between March 2015 and September 2017. Patients undergoing ORC and receiving either CTAP or TEA were included. The primary outcome was the hospital length of stay (HLOS), and secondary outcomes included time until ambulation, postoperative narcotic usage, and renal function as measured by the glomerular filtration rate (GFR).

**Results:**

We studied 146 patients, 124 of whom met our inclusion criteria. Patients receiving CTAP had a 17.4% reduction in HLOS (95% CI: 3.2, 29.4; P = 0.02) and a 13.9% reduction in time until ambulation (95% CI: 3.4, 23.3; P = 0.01) compared to those receiving TEA. This was equivalent to a relative decrease in HLOS of approximately 2.1 days in the CTAP group as compared to the TEA group. No significant differences were observed in narcotic usage or GFR between the two groups. Our sensitivity analyses using instrumental variables analysis yielded similar results.

**Conclusions:**

Continuous transversus abdominis plane was associated with a shorter HLOS and quicker time to ambulate compared to TEA, without affecting narcotic usage or renal function. These findings suggest that CTAP may be a viable alternative to TEA for perioperative analgesia in ORC patients. Further research is needed to confirm these findings.

## 1. Background

Poorly managed postoperative pain negatively affects the quality of life and functional recovery after surgery, increasing the risk of post-surgical complications and persistent postsurgical issues (1-3).

American Society of Anesthesiologists (ASA), American Society of Regional Anesthesia and Pain Medicine (ASRA-PM), and American Pain Service (APS) joint committee guidelines advise clinicians to offer multimodal analgesia to their patients during the perioperative period. The guidelines strongly recommend surgical site-specific peripheral regional anaesthetic techniques and neuraxial analgesia (1, 2, 4).

Despite this strong support for using regional anaesthesia for perioperative analgesia, its effects on extended postoperative outcomes and resource utilization are unclear. There is a growing recognition that the clinical trials in pain medicine put insufficient emphasis on measuring these outcomes, and the most recent large clinical trials on the topic show an increasing focus on such outcomes (5-8).

Open radical cystectomy (ORC), a procedure used in locally advanced bladder cancer, is an extensive abdominal surgery that requires a midline abdominal incision. During the procedure, the bladder is disconnected from the urethra and removed along with other organs and tissues. This requires a urinary diversion, typically created from loops of the intestine. The mean hospital length of stay (HLOS) in a typical ORC patient is five days or longer, and severe postoperative pain is a factor that can extend this period (9-13).

Thoracic epidural analgesia (TEA) has been commonly used in patients undergoing ORC with or without urinary diversion. Thoracic epidural analgesia can successfully reduce postoperative surgical pain and ileus in these patients. However, population-based outcome analyses suggest an increased 30-day mortality rate and a decreased likelihood of being directly discharged to home for the ORC patients receiving TEA analgesia (14).

Transversus abdominis plane (TAP) blocks can be used in patients undergoing ORC as an alternative to TEA to provide comparable analgesia. It has been shown that TAP blocks can reduce pain, opioid requirements, time to flatus, and time to bowel movement in patients undergoing radical cystectomy or other abdominal procedures (15-18).

## 2. Objectives

There is a lack of published studies comparing extended outcomes in ORC patients receiving TEA vs. TAP blocks (6). This article aims to fill this knowledge gap by comparing the two techniques regarding HLOS, time to ambulation, postoperative patient-controlled analgesia (PCA) narcotic usage, and renal function as measured by glomerular filtration rate (GFR). Our main hypothesis was that patients receiving bilateral TAP catheters would have a shorter HLOS (primary outcome of interest) than those receiving TEA. We made this hypothesis based on the findings of prior studies and systematic reviews, which reported shorter HLOS in abdominal surgery patients receiving TAP compared to those receiving TEA (14, 19).

## 3. Methods

This study was approved by the Partners Healthcare Institutional Review Board (IRB protocol # 2016P001978). The IRB waived the patient consent requirement for the study due to its retrospective nature. A retrospective chart review was performed to identify patients who underwent ORC performed by one of five surgeons at a major teaching hospital in Boston, Massachusetts, USA, between March 2015 and September 2017.

Our inclusion criteria included: (1) being 18 years of age and older; (2) undergoing ORC for advanced and invasive bladder cancer under general anaesthesia (GA) with endotracheal intubation.

Our exclusion criteria were: (1) ASA physical status class 5; (2) not getting TEA or continuous transversus abdominis plane (CTAP); (3) getting any other thoracic or abdominal wall regional anaesthesia; (4) having an allergy to local anesthetics; (5) getting any surgical procedures other than ORC; (6) contraindication to regional anaesthesia or central neuraxial anaesthesia.

Open radical cystectomy was performed through a midline incision, typically extending from T9 to L1, and the radical cystectomy was performed transperitoneally, with lymph node dissection and urinary diversion. Urinary diversions were either orthotopic neobladder, ileal conduit, or sigmoid bladder (20).

For the patients receiving TEA, the thoracic epidural placement was performed before the ORC on the preoperative day by the same anaesthesia team who cared for the patient intraoperatively. Epidurals were typically placed at the low thoracic level (T8 - T12). The infusion of local anaesthetic (LA) solution through the epidural catheter was started prior to incision and continued during the perioperative period. Postoperative pain control was provided mainly by TEA with or without PCA narcotics.

For the patients receiving CTAP, the block was performed using the standard lateral TAP approach with the placement of the catheter and injection of the local anaesthetic in the transversus abdominis plane under ultrasound guidance (21). Transversus abdominis plane catheters were placed at the end of the surgery immediately after the closure of the skin and placing of the dressing. Catheters were used immediately after placement with loading and continuous infusion of LA.

The analgesic infusion for the TEA was typically infusion of a mixture of local anesthetics (bupivacaine 0.1%) with narcotics (hydromorphone 10 micrograms per milliliter). The typical infusion rate started as 6 mL/h with patient-controlled boluses of 2 - 4 mL every 10 minutes. For the TAP catheter, the infusion was bupivacaine 0.1% with a rate of 8 - 10 mL/h per side. The Acute Pain Service followed the patients with either CTAP or TEA in the post-anesthetic care unit and daily afterward until the catheter(s) were removed.

Any additional uncovered pain will be controlled by either oral narcotics or PCA, typically hydromorphone. With the start of the systemic narcotics, the opioid component of the TEA solution will be removed, and the patient will get pure Bupivacaine 0.1% epidurally.

During the chart review, demographic and medical variables, which include sex, age, weight, and ASA physical status classification, were collected for each patient. Procedural data, including length of surgery, the total amount of IV crystalloids and colloids administered, estimated blood loss (EBL), urine output, morphine equivalent usage rate per minute during surgery (22), norepinephrine usage rate per minute during surgery, presence of nausea or vomiting, and use of intensive care unit (ICU) or post-anesthesia care unit (PACU) were also collected.

Our primary outcome was the length of hospital stay (HLOS) in days. The secondary outcomes comprised the time until ambulation (out of bed or OOB) in days, renal function trajectory defined by daily GFRs over postoperative days 0 through 7, and daily morphine equivalents usage over postoperative days 0 through 7. We also reported descriptive statistics on whether the patients required PCA, how many days of PCA they received, and the number of days until the removal of CTAP or TEA catheters for the patients in each treatment arm.

The study was powered to detect a difference of 0.5 days in HLOS with a half-day standard deviation (SD). Assuming a two-tailed alpha of 0.05 and a low missing rate (< 5%), the study would have a power of 0.80 with a sample size of N = 32.

The differences in demographic, medical, and procedural variables between the CTAP and TEA groups were reported and compared using the Welch two sample *t*-test for the continuous variables and Pearson's chi-squared test for the categorical variables. The effects of the analgesia methods (CTAP vs. TEA) on the primary and secondary outcomes were estimated using linear regression models controlling for age, sex, weight, and ASA class. We log-transformed the outcomes (outcome transformed, [log_10_(outcome) + 0.001]) for the models. Results from the log-transformed models were then back-transformed for reporting. Based on these, the resulting coefficients for the exposure (CTAP vs. TEA) and co-variates reflected the percentage differences between the groups. Estimated marginal means (EMM) were then calculated and reported to reflect the point changes in original units. We also estimated instrumental variables (IV) models as a robustness analysis to account for unmeasured confounding from factors such as unmeasured patient or surgeon characteristics. For these models, we used the year of surgery as an instrument based on an institution-wide transition from TEA to CTAP during the period of our study due to publications showing increased risks of adverse events in patients receiving TEA at the population level (14). For this instrument to be valid, we needed a substantial correlation between the instrument and the local anaesthesia method (CTAP or TEA). We confirmed this correlation using the F-statistic of the first-stage model in a 2-stage least-squares regression, which was 13.98. An F-statistic above ten is generally considered sufficient for an instrument to be valid (23). The other condition for the instrument to be valid is that there are no mechanisms other than the treatment (the local anaesthesia method) for the outcome to change based on the instrument. Although not statistically testable, we did not know of any other reasons that would change our outcomes across these years. The instrumental variable models followed the same log-transformation, back-transformation, and EMM steps as above.

Glomerular filtration rate and morphine equivalent use data over postoperative days 0 through 7 were analyzed using linear mixed-effects models (LME) with the patient identifier as the random intercept. The regressors in this model included the time as a continuous variable (i.e., trajectory) and the treatment group (CTAP or TEA). R statistical software (V3.3.2, Rstudio. Inc., Boston, MA) was used for data management and analysis. All confidence intervals were heteroskedasticity robust.

## 4. Results

**Figure 1.**
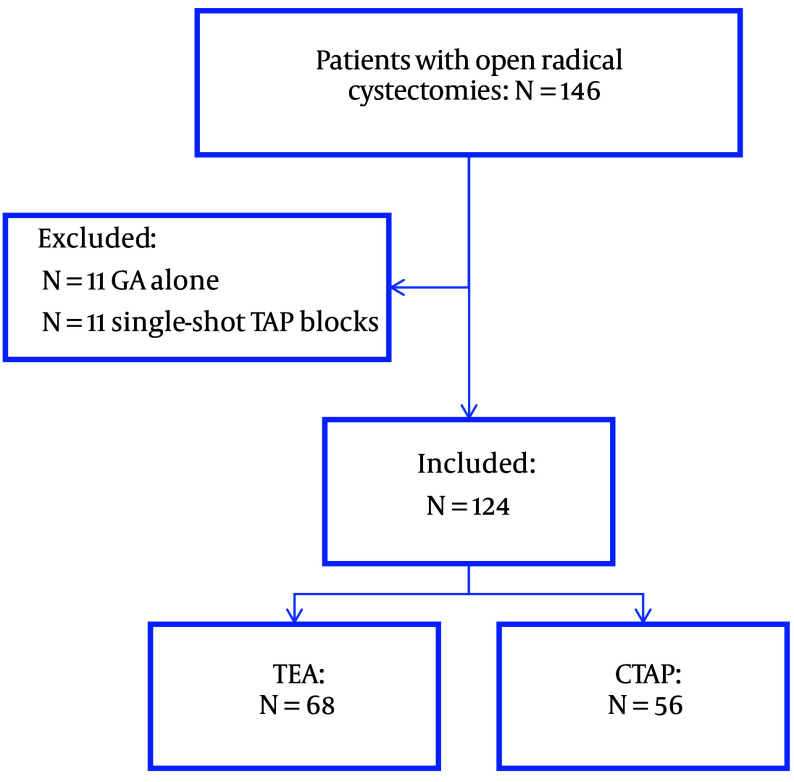
Patient chart review flow diagram

The charts of 146 patients who underwent ORC during the study period were reviewed. Eleven patients did not receive either a TAP or a TEA and hence were excluded. Furthermore, 11 patients were excluded for receiving single-shot TAP blocks rather than continuous catheters (CTAP). As a result, 124 patients who received ORC under general anaesthesia supplemented with a TEA (N = 68) or CTAP (N = 56) were included in our study (Figure 1). 

We did not find statistically significant differences between the two groups concerning age, gender, weight, and ASA class (Table 1). 

**Table 1.** Descriptive Statistics of the Demographic Variables for the Thoracic Epidural Analgesia and Continuous Transversus Abdominis Plane Receivers ^a^

Characteristics	TEA (N = 68)	CTAP (N = 56)	P-Value ^b^
**Age**	68 ± 9	67 ± 10	0.8
**Weight**	80 ± 15	82 ± 16	0.5
**Sex**			> 0.9
Female	23 (34)	19 (34)	
**ASA**			0.8
2	29 (43)	25 (45)	
3	39 (57)	31 (55)	

Abbreviations: TEA, thoracic epidural analgesia; CTAP, continuous transversus abdominis plane; ASA, American Society of Anesthesiologists Physical Status Classification.

^a^ Values are expressed as mean ± SD or No. (%).

^b^ Welch two-sample *t*-test and Pearson's chi-squared test.

Among the procedural variables, we did not find a statistically significant difference with regard to the length of surgery, the amount of IV crystalloids used for the patients, urine output, and nausea and vomiting. The amount of IV colloids, estimated blood loss, norepinephrine rate per minute of surgery, and ICU admissions were higher for the TEA patients. Conversely, morphine equivalents used per minute of surgery and PACU admissions were higher among the CTAP receivers (Table 2). 

**Table 2.** Descriptive Statistics of the Procedural Variables for the Thoracic Epidural Analgesia and Continuous Transversus Abdominis Plane Receivers^a^

Characteristics	TEA (N = 68)	CTAP (N = 56)	P-Value ^b^
**Procedure length (min)**	554 ± 117	535 ± 104	0.3
**Intraoperative IVF crystalloid infused (mL)**	7.45 ± 2.58	6.98 ± 2.65	0.3
**Intraoperative IVF colloid infused (mL)**	1.39 ± 1.60	0.74 ± 0.95	0.005
**Intraoperative EBL (mL)**	1.69 ± 1.20	1.34 ± 0.71	0.044
**Urine output (L)**	0.46 ± 0.50	0.40 ± 0.44	0.5
**Morphine equivalents per minute of surgery (mg)**	0.06 ± 0.06	0.10 ± 0.15	0.032
**Norepinephrine per minute of surgery (mg)**	23 ± 36	12 ± 7	0.014
**Nausea and vomiting**	3 (4.4)	3 (5.5)	> 0.9
**ICU vs. PACU admission**			< 0.001
PACU	47 (69)	53 (96)	
ICU	21 (31)	2 (3.6)	

Abbreviations: TEA, thoracic epidural analgesia; CTAP, continuous transversus abdominis plane; IVF, intravenous fluid; EBL, estimated blood loss; POD, postoperative day; ICU, intensive care unit; PACU, post-anesthesia care unit.

^a^ Values are expressed as mean ± SD or No. (%).

^b^ Welch two-sample *t*-test, Fisher's exact test, and; Pearson's chi-squared test.

A nasogastric tube was placed either intra- or post-operatively for a total of 38 patients (31%). The most commonly used urinary diversion technique was ileal conduit (66%), followed by orthopaedic neobladder (19%), colon loop diversion (14%), and cutaneous urethrostomy (1%).

Patients who received CTAP had a 17.0% decrease (95% CI: 2.7, 29.2; P = 0.02) in their length of stay compared to patients who received TEA. This was equivalent to a relative decrease in HLOS of approximately 2.1 days in the CTAP group as compared to the TEA group. The EMM of LOS and their 95% CIs were 11 [10, 12] in the TEA group and 9 [8, 10] in the CTAP group.

The instrumental variables analysis yielded statistically significant results in the same direction with a 35.3% decrease (95% CI: 2.1, 57.2; P = 0.04) in the length of stay for the CTAP patients compared to the TEA patients. The EMM of LOS was 12 (95% CI: 10, 15) for the TEA patients and 8 (95% CI: 6, 10) for the CTAP patients.

The CTAP group also had a 13.8% decrease (95% CI: 3.6, 23.0; P = 0.01) in OOB compared to the TEA, equivalent to a relative decrease of approximately 0.2 days. The EMM of OOB was 1 (95% CI: 1, 2) for the TEA group and 1 (95% CI: 1, 1) for the CTAP group.

Although pointing to a similar direction, the instrumental variables analysis was not statistically significant for OOB. We saw a decrease of 21.0% (95% CI: -7.51, 41.96; P = 0.13) for the CTAP patients compared to the TEA patients. The EMM of OOB was 1 (95% CI: 1, 2) for the TEA patients and 1 (95% CI: 1, 1) for the CTAP patients.

**Figure 2.**
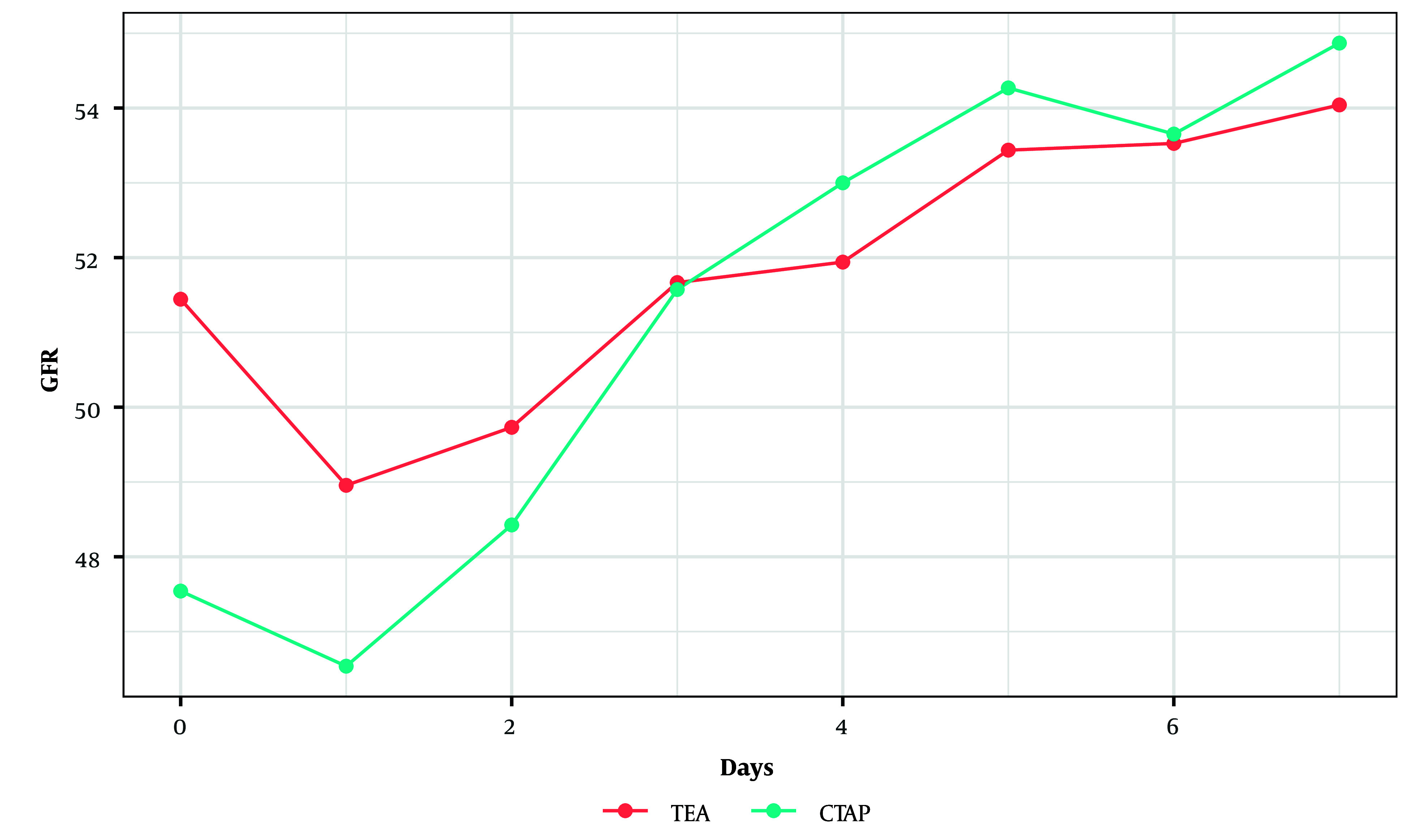
Glomerular filtration rate value changes from postoperative days 0 through 7. No significant changes were observed between the two groups.

There was no significant difference in GFR between the two groups over the postoperative days 0 to 7 (P = 0.50, linear mixed effects model) (Figure 2). 

**Figure 3.**
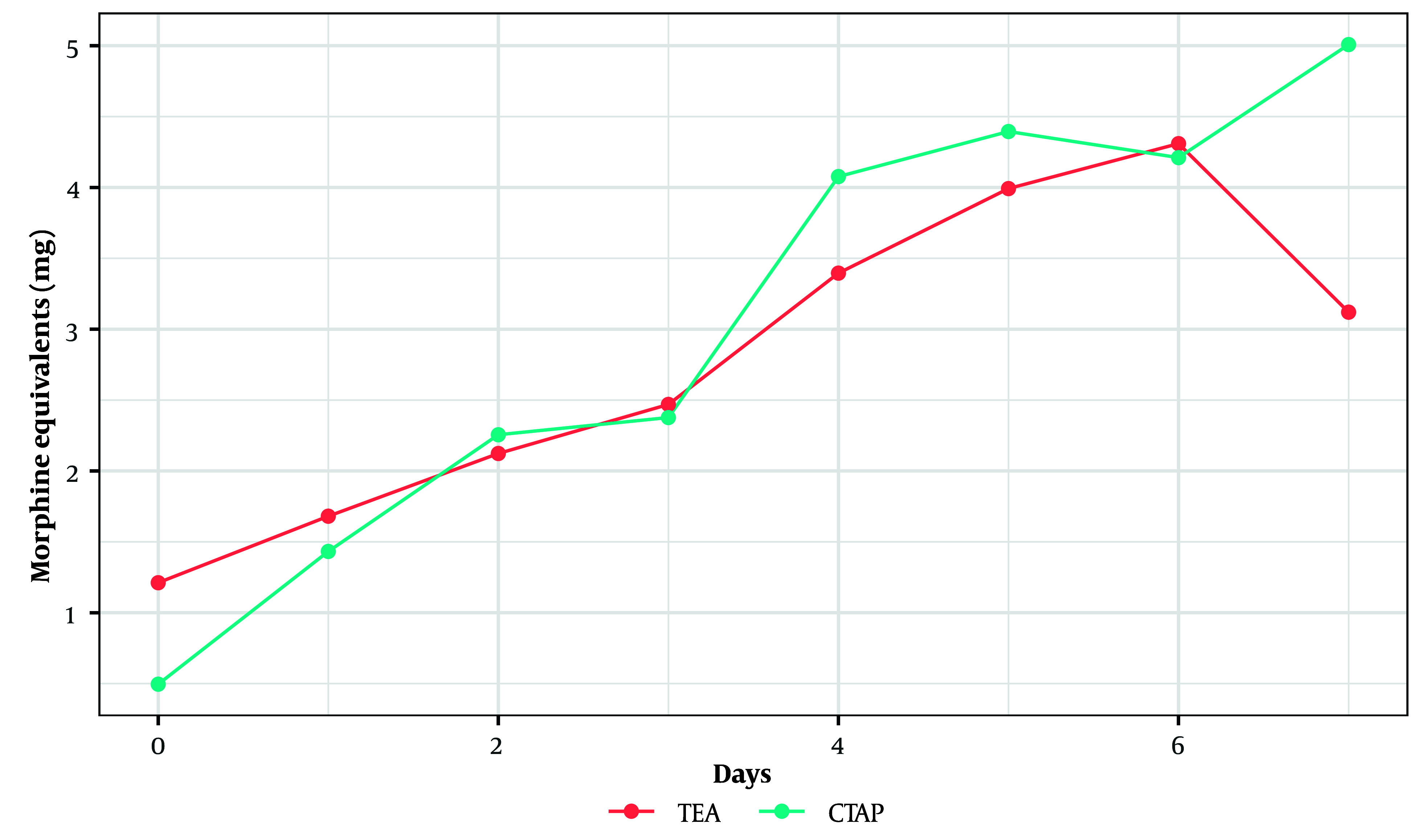
Daily morphine equivalent usage from postoperative days 0 through 7. No significant changes were observed between the two groups.

We did not find a statistically significant difference between the trajectories of morphine equivalent usage over the postoperative days 0 to 7 between the two groups (P = 0.94, linear mixed effects model) (Figure 3). 

## 5. Discussion

This study aimed to compare the effects of TEA and TAP blocks on postoperative outcomes in ORC patients. Our results showed that patients who received CTAP had a 17% decrease in their length of stay compared to patients who received TEA. The CTAP group also had a 13.8% decrease in time until out of bed compared to the TEA group. However, there was no significant difference between the two groups in GFR and morphine equivalent usage over the postoperative days 0 to 7. The instrumental variables approach corroborated our results for the primary outcome, HLOS.

For decades, epidural techniques have been considered the “gold standard” for pain management after major abdominal surgery. However, more precise assessments of the previous data and newer studies show less optimistic results (5, 8, 24, 25).

Our result regarding the decreased HLOS in the CTAP receivers was in line with the results of a study by Miller et al. In that study, the higher HLOS in the TEA receivers was attributed to the increased risk of ileus and other major complications, such as myocardial infarction in these patients (10, 14). The effect of TEA on HLOS has also been evaluated in pancreatic surgery. A retrospective review of 8098 patients who had open pancreatic surgery by Kim et al. showed that TEA was a significant predictor for the longer hospital length of stay. However, patient-reported pain scores were significantly lower in the patients receiving epidural anaesthesia than those receiving intravenous narcotics on the day of surgery (26). Furthermore, in a systemic review and meta-analysis, Baeriswyl et al. found a similar decrease in the length of stay of 0.6 days (95% CI -0.9 to -0.3 days, P < 0.001) in the TAP block receivers as compared to the TEA receivers in patients undergoing abdominal laparotomy (19). Two other meta-analyses comparing the two methods in abdominal surgery patients reported similar analgesic performances, with CTAP leading to fewer postoperative side effects (27, 28). The decreased length of stay has significant clinical relevance, as it decreases the risk of infection and medication side effects with improvement of patient outcomes. Besides, shorter hospital length of stay will improve the hospital's performance and efficacy (29).

Our study also showed that the CTAP group had ambulated slightly sooner than the TEA group. We believe this result is attributable to the possible blocking effects of the TEA on the residual motor, sensory, and sympathetic nerves, leading to muscle weakness, loss of proprioception, and hypotension in these patients (30, 31). Given the association of bed rest in surgery patients with worse outcomes, such as pulmonary complications (pneumonia and venous thromboembolism), iatrogenic weakness, delirium, gastrointestinal complications (ileus), and pressure ulcer formation, every measure promoting early mobility after abdominal surgery can potentially improve patient outcomes (31).

The main concern in the administration of TAP blocks in comparison to TEA is the quality of pain control. Although postoperative analgesia was not our primary outcome, our CTAP patients did not experience an increased postoperative narcotic requirement compared to the TEA patients, as assessed by the number of patients in the two groups who needed a PCA and the length of their use of PCA. These findings are consistent with those of Matulewicz et al., in which TAP blocks were used as a part of an enhanced recovery after surgery (ERAS) multi-modal pain control protocol in radical cystectomy. In this study, TAP use was associated with low narcotic requirements and significant improvements in time to flatus, bowel movement, and HLOS compared to traditional pain control methods, including PCA narcotics with or without local infiltration of anaesthetics or thoracic epidural blockade. However, this study did not compare TAP to any regional anaesthesia technique, including epidural analgesia (15). As mentioned above, the analgesic efficiency of the two methods was similar in two meta-analyses of abdominal surgery patients (27, 28).

Patients with urinary diversion, especially ileal conduit conversion, are at risk of renal function decline (32). The hypotension associated with TEA can hypothetically potentiate this decrease in GFR. However, our study failed to show differences in the two groups' daily GFR levels.

Our baseline procedural data showed that TEA is associated with less intraoperative narcotic usage but with the cost of increasing usage of phenylephrine. A phenomenon that is known related to sympathetic block associated with TEA. Although we found more ICU admissions in the TEA group and more PACU admissions in the CTAP group, our study is not powerful enough to make a meaningful conclusion.

The results of this retrospective study should only be interrupted in the context of its limitations. Retrospective studies are prone to different biases, including selection and recall bias. Using our electric medical record, we reduced the selection and the recall bias by carefully selecting the population and accurately accessing the data. The data were reassessed by multiple research members to reduce the recall bias even further. It was difficult to control all the confounding variables in a retrospective study. Multiple postoperative events can potentially prolong the HLOS, which can’t be effectively controlled for in this retrospective study and may not be adequately controlled for with the included covariates. While our statistical methods were used to adjust for confounding, it is not always possible to account for all potential confounders.

The transition from TEA to CTAP was mainly based on the request from the urology team and the agreement with the anesthesiology team regarding the effect of the TEA on HLOS and complications associated with it in ORC patients (33). The contraindications between the two regional anaesthesia methods are similar in our institute, and the methods were mostly chosen as equal alternatives. We controlled for ASA status in our models, which can account for the differences in the overall health condition between the two groups and did not reveal any significant difference.

Furthermore, we detected some differences regarding the procedural variables between the two groups (Table 2). Although these differences might raise concerns regarding the comparability of the two groups, our instrumental variables analysis could account for such differences, as none of them are expected to be related to the year of surgery. Also, although our study showed a decrease in HLOS, this may not necessarily translate to a decrease in the cost of hospitalisation.

Despite these limitations, our study provides strong evidence regarding the advantages of CTAP in HLOS and other outcomes compared to TEA.

It should be mentioned that the assumption of our instrumental variables regarding the lack of changes in the outcome across the years based on factors other than the use of TEA vs. CTAP is not testable. While the authors do not believe any such changes happened during this period, it is nonetheless a limitation. Our instrumental variables analysis is intended to be a sensitivity analysis, supplementing the results of our main models. The concordance between the results of our main models and our instrumental variables models gives us a higher confidence in our findings. Nevertheless, our study cannot take the place of a prospective randomized controlled trial. Rather, it is intended as a stepping stone, providing evidence on this less-studied subject in order the pave the way for such future prospective studies.

Furthermore, this study was conducted in a large academic hospital with substantial resources and expertise to perform either CTAP or TEA procedures. Our results might not be directly generalizable to other settings, especially if the resources or expertise in any of these methods are not similar to our setting.

We did not have any report of any adverse event or complication associated with CTAP or TEA. Adverse events and especially severe complications associated with these two regional anaesthesia/analgesia methods need a larger sample size.

In conclusion, Using CTAP for post-ORC pain control may reduce the patient's hospital length of stay and shorten the time to ambulation compared to TEA without compromising pain control. Further studies using a prospective randomised controlled trial design investigating the differences in outcomes between the patients receiving peripheral and neuraxial analgesia are warranted to further clarify the differences between the two.

## Data Availability

The protocols for the research and the associated data set used and/or analyzed during this study are available from the corresponding author upon reasonable request.
